# Optical Simulation Design of a Short Lens Length with a Curved Image Plane and Relative Illumination Analysis

**DOI:** 10.3390/mi15010064

**Published:** 2023-12-28

**Authors:** Wen-Shing Sun, Chuen-Lin Tien, Yi-Hong Liu, Guan-Er Huang, Ying-Shun Hsu, Yi-Lun Su

**Affiliations:** 1 Department of Optics and Photonics, National Central University, Taoyuan 32001, Taiwan; wssun@dop.ncu.edu.tw (W.-S.S.); yihongluis@gmail.com (Y.-H.L.); gehuang@dop.ncu.edu.tw (G.-E.H.); dmcandymurray@gmail.com (Y.-S.H.); a0357841@gmail.com (Y.-L.S.); 2 Department of Electrical Engineering, Feng Chia University, Taichung 40724, Taiwan; 3 National Applied Research Laboratories, Taiwan Instrument Research Institute, Hsinchu 300092, Taiwan

**Keywords:** optical design, curved image plane, relative illumination, Seidel aberration, Code V software

## Abstract

This study proposes a three-lens design with a short lens length and explores the curved imaging plane and performs a relative illumination analysis. There are two ways to reduce the lens length: shortening the back focal and lens group lengths. We derived the relevant parameter relationships of three lenses using the first-order geometric optics theory. The optical lens length can be controlled within 2 mm. The shorter the lens length, the larger the angle of the chief ray in the image space, resulting in an increase in the field curvature and astigmatism. Third-order Seidel aberrations can be effectively reduced by a curved image plane. We also derived the equations for relative illuminance, solid angle, surface transmittance, and internal transmittance for the short three-lens design. The optical lens design uses a curved image plane to shorten the distance from the off-axis beam image space to the image plane and reduce the incident angle of the chief ray on the image plane. The formula and design results verified by Code V software (version 11.2) show that both the solid angle and relative contrast of the lens can be increased. For the proposed three-lens design with a short lens length, the semi-field angle is 32°, F/# is 2.7, the effective focal length is 1.984 mm, the image plane area is 2.16 mm × 1.22 mm, and the curvature radius of the concave image plane is 3.726 mm. Moroever, the MTF (100 lp/mm) is larger than 52%, the lateral color aberration is less than 2.12 μm, the optical distortion is less than 2.00%, and the relative illumination is greater than 68%.

## 1. Introduction

In recent years, more and more research has focused on the advantages of curved sensors, which can reduce the required lens length and improve the image quality compared to planar sensors. The effects of a curved sensor based on the Seidel aberration theory were analyzed, and a new way to design an optical system with a curved sensor was proposed [1]. Gaschet et al. showed the entire process to create curved sensor-based optical systems and their possibilities, and discussed how the curved focal plane shape was related to the imaged scenes and optical parameters [2]. For instance, changing the primary detection surface from a flat to a curved surface within an optical system and accurately matching the applied CCD shape to the contours of the curved focal plane significantly increased the amount of transmittable light at various wavelengths through the system. Swain [3] revealed the application of curved detectors in astronomical telescopes and believed that curved focal plane detection would improve the optical performance of reflecting telescopes, achieving higher image sensitivity, lower optical aberrations, a wider field of view, and greater versatility and simplicity. The optical design requires fewer optical surfaces. Rim [4] reported using a ball lens with a spherically curved image plane and a curvature radius of 5.87 mm. Their analysis showed that the curved image provided a large degree of freedom in the design of a camera system, helped reduce the fundamental aberrations, and provided a better resolution and brightness. Iwert [5] demonstrated important developments in the field of curved surface detectors and proposed an application design for a large-sized (90 mm square) concave spherical surface detector with a radius of curvature of 250 to 500 mm.

Dumas [6] designed a 10 mm × 10 mm curved focal plane detector with a concave surface curvature radius of 40 mm. He showed that the curved focal plane had better contrast and relative illumination properties than a planar focal plane. Itonaga et al. [7] also developed a curved CMOS image sensor. They found the central sensitivity and marginal sensitivity of the curved focal plane to be 1.4 and 2 times that of the planar focal plane sensor, respectively. Reshidko [8] discussed the benefit of using curved imaging surface lenses in a miniature mobile camera, demonstrating that the curved sensor technology allowed for optically faster lens solutions. Chen [9] proposed a patent for a curved focal plane design, which incorporated three aspheric lenses and a curved focal plane with a concave surface with a curvature radius of 1.364 mm, for the implementation of a 0.92 megapixel lens. The lens length was 1.95 mm, F/# = 1.6, entrance pupil aperture D = 0.75 mm, with a lens focal length of f = 1.2 mm. Guenter et al. [10] obtained a patent for 13 different curved focal plane designs while working for Microsoft in 2016. From two to four lenses, the minimum lens length was 9.76 mm, the radius of curvature of the bent sensor was 3.623 mm, and the diameter was 3.586 mm. Gaschet et al. [11] described the benefits of incorporating curvature and tunable curvature into an existing fish-eye lens. They fabricated a surface sensor, 37 mm × 30 mm in size, with a sensor curvature radius of 150 mm. In 2019, Gaschet et al. [2] further developed curved image sensor systems. They proposed two curved image surface lens designs: one using a CMOS sensor with a fully convex surface curvature radius of 280 mm, a wide angle with a full-field of 180°, and an aperture size of F/4; the other one used a 1.3 megapixel 1/1.8” concave CMOS sensor, with the following lens specifications: an aperture size of F/2.6 and full-field angle of 40°. The optical design included six lenses, with a lens length from the vertex of the first plane to the image surface of 10 mm. They also investigated the effects of any potential defects of a curved sensor on the optical system. The tolerance process of curved sensors and their inclusion in optical designs were demonstrated. The effects of a curved sensor based on the Seidel aberration theory were analyzed, and a new path to design an optical system with a curved sensor was proposed [12].

This study proposes a three-lens design with a short lens length and curved image plane. To achieve such a short lens length, the lens group and back focal lengths of the lens system must be reduced. Moreover, the equations for relative illuminance, solid angle, surface transmittance, and internal transmittance used for the short three-lens design are presented in this work. The lens group length of the proposed optical system from the first surface to the last surface can be shortened, resulting in a lens length of 2 mm. The achievable target specifications for designing three lenses with ultra-short lens lengths are as follows. The half field angle within the lens is 32°, the radius of curvature of the concave image plane is 3.726 mm, and the relative illumination can be increased to 68%.

## 2. Methodology

### 2.1. Lens Length Definition

**Figure 1:**
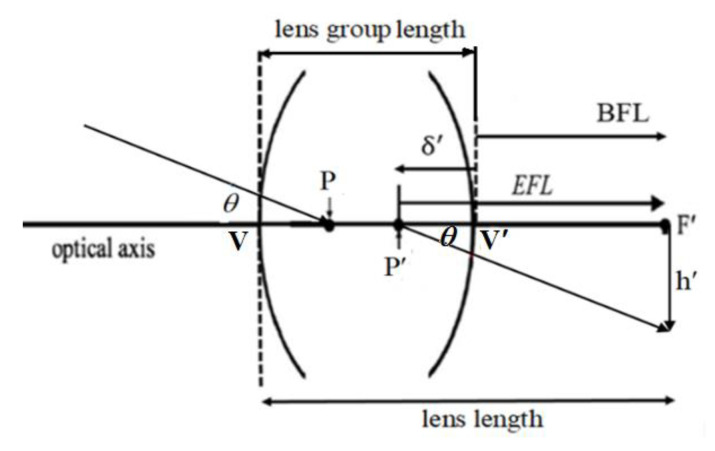
Definition of the lens length.

The lens length was defined as the distance from the vertex of the first surface of the lens to the image plane. Figure 1 illustrates the definition of the lens length when the object is at infinity and the lens is placed in the air. Here, P and P’ indicate the first and the second principal points, respectively; V and V’ are the vertex points of the first and last surfaces of the lens; F’ is the image focal point; and θ is the half field angle. The effective focal length (EFL) is the distance between P’ and F’, back focal length (BFL) is the distance from V’ to F’, and δ’ is the distance from V′ to P′. The relationship between BFL, EFL, and δ’ is as follows:

(1) BFL=EFL + δ’

When describing an optical system, a sign convention was used to distinguish the relative relationship. In this case, the direction to the left was negative and the direction to the right was positive, so δ′ was negative; BFL and EFL were both positive. Furthermore, the up direction was positive and the down direction was negative, so h’ (image height) was negative. The angle sign was positive in the clockwise direction from the ray trace to the optical axis, and negative in the counterclockwise direction from the ray trace to the optical axis, so θ was negative. The relationship between θ, h’, and EFL was EFL = h′/tanθ [13].

The lens length was the length of VF′, which meant VV′ (lens group length) plus V′F′ (BFL). The lens length value can reduce the BFL and lens group length; then, the lens length can be decreased. To reduce the BFL, the negative length of δ′ can be increased. If the lens group length needs to be decreased, the number of lens elements must be reduced. A single lens cannot correct chromatic aberrations, while a two-element lens group can correct chromatic aberrations but cannot correct large-angle aberrations. Considering 7 kinds of Seidel aberrations, and that 3-lens design has 8 degrees of freedom, which can correct 7 kinds of aberrations with a fixed-lens focal length, we chose an optical design of 3 lenses for a 1/2 viewing angle of 32° and an F # of 2.7. The thickness of the three lenses and the spacing between the lenses should be small, and aspherical lenses should be used to increase the quality of the lenses.

### 2.2. First-Order Lens Design

#### 2.2.1. First-Order Design of a Single Lens

**Figure 2:**
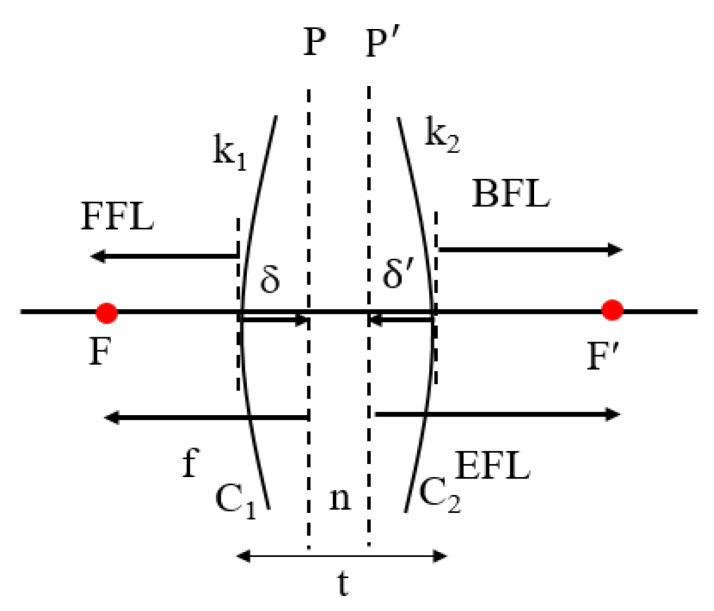
Design parameters for a single lens.

The optical design for different parameters of a single lens is shown in Figure 2. C_1_ and C_2_ indicate the curvatures of the first and second lens surfaces, k_1_ and k_2_ are the powers of the first and second lens surfaces, and F and F’ are the object and image focal points, respectively. The refractive index of the lens is n and t stands for the center thickness of the lens, while the front focal length (FFL) is the distance from the vertex of the first surface of the lens to F. The object focal length (f) is the distance from the first principal point, P, to F, and δ is the distance from the vertex of the first surface of the lens to P.

As shown in Figure 2, we set k_1_ = (n − 1)C_1_, k_2_ = (1 − n)C_2_, and k is the power of the lens; then:

(2) $$k=k_{1}k_{2}-\frac{t}{n}k_{1}k_{2},$$

where EFL, BFL, and δ′ are formulated as follows:

(3) $$\mathrm{EFL}=\frac{1}{k},$$

(4) $$\mathrm{BFL}=\frac{(1-\frac{t}{n}k_{1})}{k},$$

(5) $$\delta ’=\mathrm{BFL}-\mathrm{EFL}=-\frac{t}{n}\frac{k_{1}}{k},$$

The equations for f, FFL, and δ are defined as follows:

(6) $$f=-\frac{1}{k},$$

(7) $$\mathrm{FFL}=-\frac{(1-\frac{t}{n}k_{2})}{k},$$

(8) $$\delta =\mathrm{FFL}-f=\frac{t}{n}\frac{k_{2}}{k}.$$

#### 2.2.2. First-Order Design of the Three Lenses

**Figure 3:**
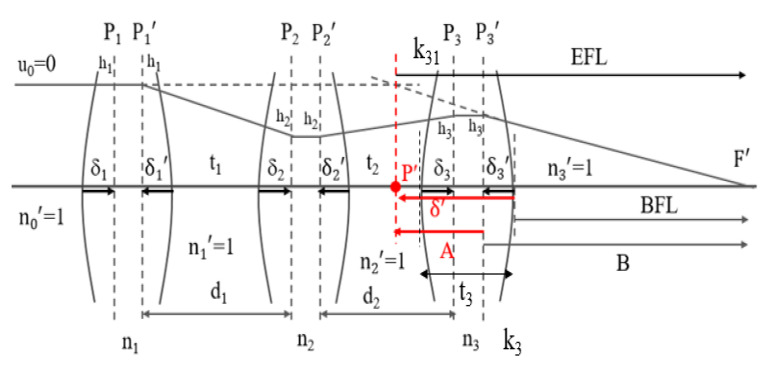
First-order design of the three-lens group.

The proposed lens design comprised three lenses, as shown in Figure 3. The symbols P_1_, P_1_’, P_2_, P_2_’, P_3_, and P_3_’ are defined as the first and second principal points of the first, second, and third lenses. k_1_, k_2_, and k_3_ are the refractive powers of the first, second, and third lenses, respectively. k_31_ is the refractive power of the first surface of the third lens, and k is the refractive power of the three-lens group. H_1_, h_2_, and h_3_ are the heights of the marginal ray on the principal plane of the first, second, and third lenses, respectively. Here, t_1_ is the distance from the vertex of the second surface of the first lens to the vertex of the first surface of the second lens, while t_2_ is the distance from the vertex of the second surface of the second lens to the vertex of the first surface of the third lens. The lens distance signs include positive and negative: negative to the left, and positive to the right. δ_1_, δ_2_, and δ_3_ indicate the distances from the vertex of the first surface to the first principal planes of the first, second, and third lenses, respectively; δ_1_’, δ_2_′, and δ_3_′ indicate the distances from the vertex of the second surface to the second principal planes of the first, second, and third lenses, respectively; d_1_ is the distance from the second principal plane of the first lens to the first principal plane of the second lens; and d_2_ is the distance from the second principal plane of the second lens to the first principal plane of the third lens.

d_1_ = −δ_1_′ + t_1_ + δ_2_, (9)

d_2_ = −δ_2_′ + t_2_ + δ_3_. (10)

In addition, BFL_3_ is the back focal length of the third lens and EFL_3_ is the effective focal length of the third lens. t_3_ is the center thickness of the third lens. n_3_ is the refractive index of the third lens. k_31_ is the refracting power of the first surface for the third lens and k_3_ is the refracting power of the third lens. Thus, the distance, δ_3_′, from the vertex of the second surface to the second principal plane of the third lens is given by:

(11) $${\delta ’}_{3}={\mathrm{BFL}}_{3}{-\mathrm{EFL}}_{3}=-\frac{t_{3}}{n_{3}}\frac{k_{31}}{k_{3}}$$

where BFL is the back focal length of the three-lens group. EFL is the effective focal length of the three-lens design (EFL = 1/k). B is the distance from the second principal plane of the third lens to the image space focus point of the three lenses. A is the distance from the second principal plane of the third lens to the second principal point of the three-lens group.

(12) $$B=\frac{{(1-d}_{1}k_{1}-d_{2}k_{12})}{k},$$

(13) $$A=B-\mathrm{EFL}=-\frac{{(d}_{1}k_{1}+d_{2}k_{12})}{k},$$

where k_12_ is the refracting power of the group of the first and second lenses, and k_12_ = k_1_ + k_2_ − d_1_k_1_k_2_. Therefore, the distance, δ′, from the vertex of the last surface to the second principal plane of the three-lens group is expressed as:

(14) $$\delta ’=A+\delta_{3}’=-\frac{{(d}_{1}k_{1}+d_{2}k_{12})}{k}-\frac{t_{3}}{n_{3}}\frac{k_{31}}{k_{3}}$$

### 2.3. Relative Illumination

The relative illuminance value is related to the solid angle on the imaging surface, the surface transmittance, and the internal transmittance of the lens system.

#### 2.3.1. Definition of Solid Angle

The definition of a circular solid angle is as follows:

(15) $$\Omega =\pi n_{0}^{2}\sin^{2}\theta_{0}=\pi {\mathrm{NA}}^{2},$$

where n_0_ is the refractive index in the medium, θ_0_ is the angle between the marginal and chief rays, and *NA* is the numerical aperture.

If the angle, θ_X_, between the marginal ray in the horizontal direction and the chief ray is different from the angle, θ_Y_, between the marginal ray in the vertical direction and the chief ray, then the solid angle is an elliptical solid angle, which can be expressed as follows:

(16) $$\Omega =\pi n_{0}^{2}\sin\theta_{X}\sin\theta_{Y}=\pi {\mathrm{NA}}_{X}NA_{Y},$$

where NA_X_ and NA_Y_ are the numerical apertures in the horizontal and vertical directions, respectively.

**Figure 4:**
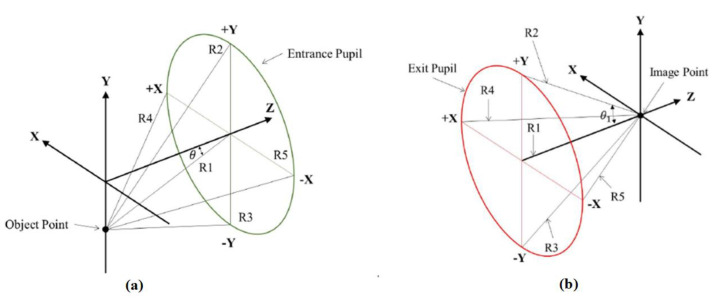
Reference rays: (**a**) definition of reference ray; (**b**) on-axis reference ray imaging distribution.

Five reference rays, R1, R2, R3, R4, and R5, were defined using the Code V optical software [14], as shown in Figure 4. In Figure 4a, R1, which is the chief ray, enters the center point of the entrance pupil. The angle, θ, between the chief ray in the object space and optical axis is the semi-field angle. R2 indicates the marginal ray, which enters normally above the entrance pupil (+Y); R3 is the marginal ray, which enters normally under the entrance pupil (−Y); R4 is the marginal ray, which enters normally to the left of the entrance pupil (+X); and R5 is the marginal ray, which enters normally to the right of the entrance pupil (−X). The semi-field angle is θ = 0°. The five reference rays, R1, R2, R3, R4, and R5, exit onto the image plane, as shown in Figure 4b, where the angles, θ_1_, between the marginal rays (R2, R3, R4, and R5) and chief ray (R1) are all the same. The image space on-axis numerical aperture, *NA*_0_, is:

(17) $${\mathrm{NA}}_{0}=n\sin\theta_{1},$$

where *n* is the refractive index in the image space medium.

**Figure 5:**
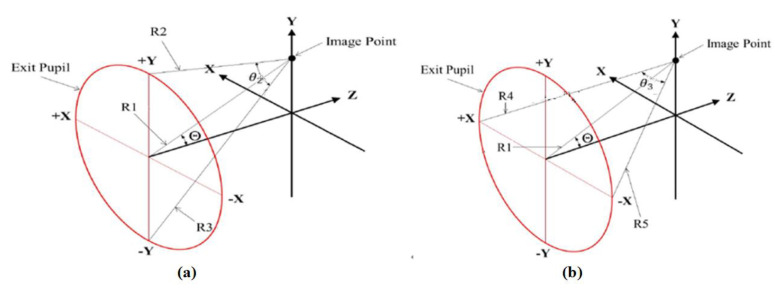
Reference rays on image plane: (**a**) Y-direction reference rays; (**b**) X-direction reference rays.

The reference rays on the image plane when the semi-field angle is θ ≠ 0° are shown in Figure 5. Figure 5a shows reference rays R1, R2, and R3 in the Y-direction. The three rays are coplanar, where the different directional cosines for R1, R2, and R3 are R1(L_1_, M_1_, N_1_), R2(L_2_, M_2_, N_2_), and R3(L_3_, M_3_, N_3_), respectively. The directional cosine is the unit vector, where Θ is the angle between the chief ray and the optical axis in the image space. If θ_2_ is the angle between R2 and R3, then:

(18) $$\begin{array}{cc} \theta_{2} & =\cos^{-1}\left(\frac{L_{2}L_{3}+M_{2}M_{3}+N_{2}N_{3}}{\sqrt{{L_{2}}^{2}+{M_{2}}^{2}+{N_{2}}^{2}}\sqrt{{L_{3}}^{2}+{M_{3}}^{2}+{N_{3}}^{2}}}\right)\\ & =\cos^{-1}\left(L_{2}L_{3}+M_{2}M_{3}+N_{2}N_{3}\right) \end{array}.$$

The numerical aperture, NA_Y_, in the Y-direction is expressed as follows:

(19) $$NA_{Y}=n\sin\frac{\theta_{2}}{2}.$$

As R1, R4, and R5 are not coplanar in the X-direction, but R4 is coplanar with R5, then, if θ_3_ is the angle between R4 and R5, the equation for finding θ_3_, as shown in Figure 5b, is:

(20) $$\theta_{3}=\cos^{-1}\left(L_{4}L_{5}+M_{4}M_{5}+N_{4}N_{5}\right).$$

The numerical aperture NA_X_ in the X-direction is expressed as:

(21) $$NA_{X}=n\sin\frac{\theta_{3}}{2}.$$

**Figure 6:**
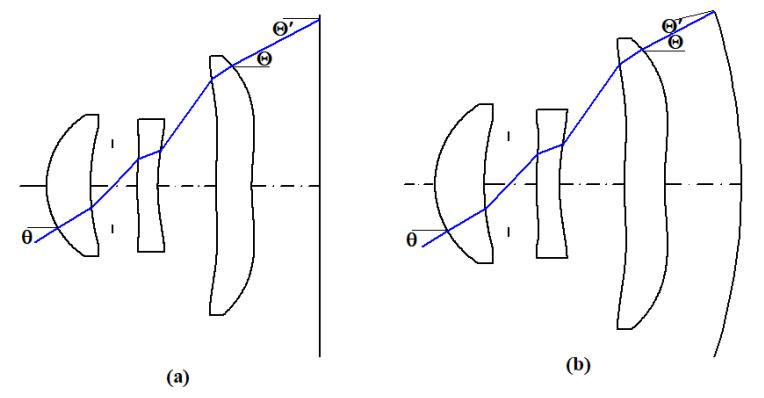
Relation of the incident angle to the distance between the chief ray and image plane for the (**a**) planar image plane and (**b**) curved image plane.

The design of the same lens on a planar and a curved image plane is shown in Figure 6. Figure 6a shows the incident angle and distance relation of the chief ray on the planar image plane when the semi-field angle is θ ≠ 0°. It can be seen that, when the chief ray trace enters the planar image plane, where Θ is the exit angle of the chief ray in the image space and Θ’ is the incident angle of the chief ray on the planar image plane, Θ’ = Θ. As can be seen in Figure 6b, when the chief ray trace enters the curved image plane, the exit angle, Θ, of the chief ray is not equal to the incident angle, Θ′, on the curved image plane, i.e., Θ’ ≠ Θ, and Θ’ < Θ. The distance covered by the chief ray in the image space from the last plane of the lens to the curved image plane is shorter than the distance to the planar image plane. The numerical apertures NA_X_ and NA_Y_ create a sinusoidal angle between the marginal and chief rays in the image space; thus, when the exit pupil aperture (D_ex_) is fixed, the shorter the distance from the chief ray to the image plane, the larger the numerical apertures NA_X_ and NA_Y_ will be. Therefore, the curved image plane design has larger NA_X_ and NA_Y_ values than the planar image plane design.

**Figure 7:**
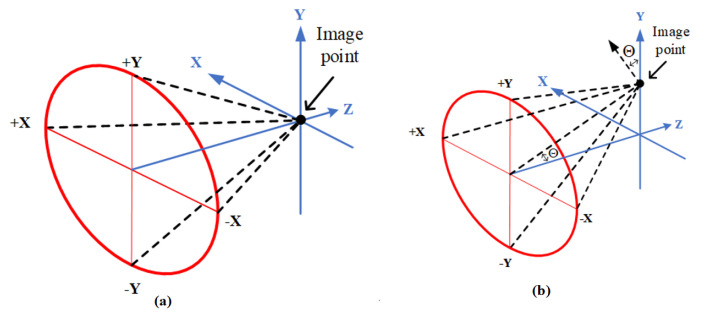
Ordinary and projector solid angles: (**a**) ordinary solid angle Ω_θ=0_. is a circular solid angle; (**b**) ordinary solid angle Ω_θ_ is an elliptical solid angle, and the projected solid angle is Ω_θ_′; then, Ω_θ_′ = Ω_θ_cosΘ.

The solid angles on the image plane are classified as either ordinary or projected solid angles, as shown in Figure 7.

When the semi-field angle is θ = 0°, the chief ray is imaged at the center point of the image planar plane on the optical axis, and the chief ray is perpendicular to the image plane, meaning that the angle of the chief ray, Θ, in the image space is 0°, as shown in Figure 7a, and the ordinary solid angle is Ω_θ=0_. The ordinary solid angle is symmetric, that is, a circular solid angle. When the semi-field angle is θ ≠ 0°, the image of the chief ray appears at some height on the image plane. The ordinary solid angle, Ω_θ_, becomes asymmetric, that is, an elliptical solid angle. The angle between the chief ray in the image space and the optical axis is Θ, and the incident angle of the chief ray on the planar image plane is Θ. The ordinary solid angle, Ω_θ_, projected in the vertical direction of the image plane is defined as projected solid angle Ω_θ_′; then, Ω_θ_′ = Ω_θ_cosΘ, as shown in Figure 7b. If the image plane is a curved image surface and the incident angle of the chief ray on the planar image plane is Θ′, then Θ′ ≠ Θ. The projected solid angle Ω_θ_′ is Ω_θ_′ = Ω_θ_cosΘ′.

#### 2.3.2. Surface Transmittance

An anti-reflection thin film was coated on the surface of the lens to improve the surface transmittance. The transmittance of light on the surface was different for S-polarized light and P-polarized light. In this paper, the surface of each lens was coated with a layer of magnesium fluoride (MgF_2_), which had a refractive index of 1.38, the optical thickness of the quarter central wavelength (λ/4). The S-polarized light reflectance [15] is:

(22) $$R_{S}={\left|\frac{An_{0}\cos\theta +Bn_{S}n_{0}\cos\theta_{S}\cos\theta -C-Dn_{S}\cos\theta_{S}}{An_{0}\cos\theta +Bn_{S}n_{0}\cos\theta_{S}\cos\theta +C+Dn_{S}\cos\theta_{S}}\right|}^{2},$$

where n_S_ is the refractive index of the lens; n_0_ is the refractive index of air; θ is the incident angle of the chief ray of the center wavelength from the air into the thin film layer; and the ABCD matrix is a single-layer film transfer matrix, as shown in Equation (23):

(23) $$M=\left[\begin{array}{cc} A & B\\ C & D \end{array}\right]=\left[\begin{array}{cc} \cos\alpha & -\frac{i}{p}\sin\alpha \\ -ip\sin\alpha & \cos\alpha \end{array}\right],$$

where α = klcosβ and p = ncosβ; k is the wave number of the reference wavelength light; l is the path length of the chief ray of the reference wavelength light in the film; n is the refractive index of the magnesium fluoride film; and β is the angle of the chief ray of the reference wavelength light in the film. The P-polarized light reflectance [15] is as follows:

(24) $$R_{P}={\left|\frac{-An_{0}\cos\theta_{S}-Bn_{S}n_{0}+C\cos\theta_{S}\cos\theta +Dn_{S}\cos\theta }{An_{0}\cos\theta_{S}+Bn_{S}n_{0}+C\cos\theta_{S}\cos\theta +Dn_{S}\cos\theta }\right|}^{2},$$

where ABCD is a single-layer film transfer matrix, as shown in Equation (23), and α = klcosβ and p = n/cosβ. If the light is unpolarized, the surface reflectance of the unpolarized light is the average of the reflectance of the S-polarized (RS) and P-polarized (RP) lights; R_θ_ = (R_S_ + R_P_)/2. The surface transmittance is ST_θ_; ST_θ_ = 1 − R_θ_.

#### 2.3.3. Internal Transmittance

The influence of the surface transmittance was not considered in the internal transmittance. The internal transmittance of the lens was τ_d_, and a decrease in transmittance was caused by the absorption inside the lens.

The value of τ_d_ was related to the material and thickness of the lens. The SCHOTT glass company provided internal transmittance data for only 10 and 25 mm thick lenses at different wavelengths. The internal transmittance for lenses of different thicknesses were obtained, assuming that the half field angle, θ, was zero, the path length, d_0_, of the chief ray in the lens and its internal transmittance was τ_d0_, while the other half field angle, θ, took the path length, d_θ_, in the lens of the same material. The internal transmittance, τ_dθ_, can be calculated by Equation (25) [16]:

(25) $$\tau_{d_{\theta }}={\tau_{d_{0}}}^{\left(d_{\theta }/d_{0}\right)}.$$

#### 2.3.4. Relative Illumination Equation

The relative illuminance was defined as the ratio of the illuminance at any point to the illuminance at the center point on the image plane; so, the projected solid angle of the light converged on the image surface under different viewing angles.

If the exit pupil surface was Lambertian, when the half field angle θ = 0°, the luminance emitted at the exit pupil was $L_{0}$ , its solid angle $\Omega_{0}$ was a circular solid angle, and the illuminance at the center point of the image surface was $E_{0}$ . Similarly, when θ was the maximum half field angle, the luminance emitted at the exit pupil surface was $L_{\theta }$ , L_θ_ = L_0_, its solid angle, $\Omega_{\theta }^{’}$ , was the ellipse solid angle, and the illuminance at the edge of the image surface was $E_{\theta }$ .

The illumination equations for the optical system are listed below:

(26) $$E_{0}=L_{0}\Omega_{0}\prod_{i=1}^{n}ST_{0_{i}}\prod_{i=1}^{m}\tau_{d_{0_{i}}},$$

(27) $$E_{\theta }=L_{\theta }{\Omega^{\prime }}_{\theta }\prod_{i=1}^{n}ST_{\theta_{i}}\prod_{i=1}^{m}\tau_{d_{\theta_{i}}}=L_{\theta }\Omega_{\theta }\cos\Theta^{\prime }\prod_{i=1}^{n}ST_{\theta_{i}}\prod_{i=1}^{m}\tau_{d_{\theta_{i}}},$$

where n is the total number of lens surfaces, n = 6; m is the total number of lenses, m = 3. The relative illumination is expressed in Equation (28):

(28) $$RI=\frac{E_{\theta }}{E_{0}}=\frac{NA_{Y}NA_{X}\cos\Theta^{\prime }(\prod_{i=1}^{n}{\mathrm{ST}}_{\theta_{i}})(\prod_{i=1}^{m}\tau_{d_{\theta_{i}}})}{{\left(NA_{0}\right)}^{2}(\prod_{i=1}^{n}{\mathrm{ST}}_{0_{i}})(\prod_{i=1}^{m}\tau_{d_{0_{i}}})}.$$

The relative illumination, RI, represented by the angle between the marginal and chief rays on the image plane is expressed as follows:

(29) $$RI=\frac{\sin\frac{\theta_{2}}{2}\sin\frac{\theta_{3}}{2}\cos\Theta^{\prime }(\prod_{i=1}^{n}{\mathrm{ST}}_{\theta_{i}})(\prod_{i=1}^{m}\tau_{d_{\theta_{i}}})}{\sin^{2}\theta_{1}(\prod_{i=1}^{n}{\mathrm{ST}}_{0_{i}})(\prod_{i=1}^{m}\tau_{d_{0_{i}}})}.$$

## 3. Design Results

For the curved image plane lens design, a two-megapixel sensor from Toshiba T4K71 was used in the curved image plane simulation. The effective area was 2.159 mm × 1.219 mm and the pixel size was 1.12 μm × 1.12 μm. The proposed three-lens design had three aspherical lenses with six aspherical surfaces. The aspheric surfaces can be defined by the following formula:

(30) $$Z\left(r\right)=\frac{C_{v}r^{2}}{1+\sqrt{1-\left(1+K\right)C_{v}^{2}r^{2}}}+Ar^{4}+Br^{6}+Cr^{8}+Dr^{10},$$

where Z(r) is the sag of the surface parallel to the optical axis (at distance r from the axis); r is the radial distance from the optical axis; $C_{v}$ is the curvature, the inverse of the radius of curvature; K is the conic constant; and A, B, C, and D indicate the fourth-, sixth-, eighth-, and tenth-order aspheric coefficients, respectively.

**Figure 8:**
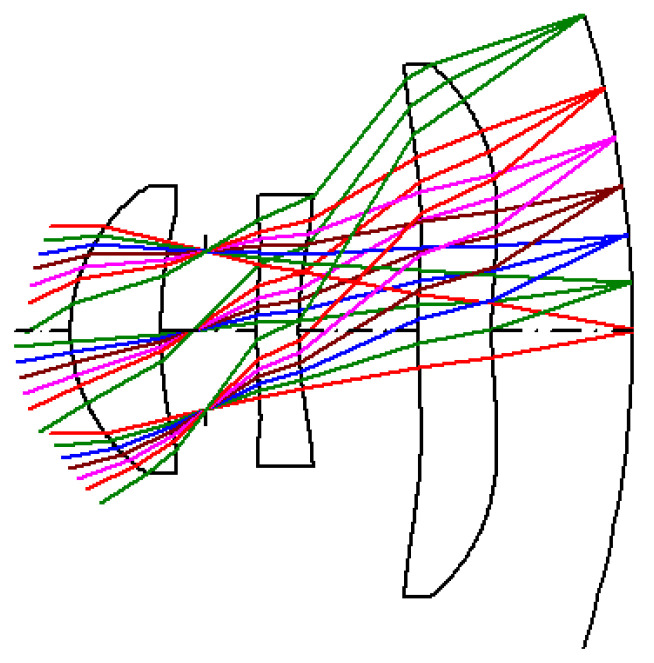
Three-lens design with a curved image plane.

The lens design data and the values of the aspherical coefficients of the three-lens design are shown in Table 1 and Table 2, respectively. The three-lens layout with the curved image plane is shown in Figure 8. The effective focal length (EFL) is 1.984 mm, the half field angle is θ = 32°, and the image height is h = 1.2397 mm.

### 3.1. Lens Length

The lens length is the lens group length plus BFL. In this work, the lens design was created by using Code V optical software, and the optimization settings mainly focused on a lens length = 2 mm and a BFL ≤ 0.5 mm. At the beginning, it was difficult to meet the requirements for the optimization. When considering the first-order design of the lens length and the optimized lens design, the δ′ negative length must be increased and the lens length must be shortened, since the length of δ′ is expressed as in Equation (14) and the values and optimizations of d_1_, d_2_, k_1_, k_12_, k_31_, k_3_, t_3_, and n_3_ are controlled. Therefore, during the optimization process, it was easy to increase the negative value, δ′, resulting in a decrease in the BFL, a decrease in the lens length, and a decrease in the relative lens group length. Finally, a three-lens design with a lens length of 2 mm, BFL of 0.5 mm, and a length group length of 1.5 mm was obtained. When the second principal plane position (P′) of the system was moved closer towards the object space, as shown in Table 3, then δ′ = −1.48405 mm. The lens group length was shortened to 1.5 mm and the lens length was shortened to 2.00 mm.

### 3.2. Relation Illumination

The relative illuminance was related to the projected solid angle, surface transmittance, and internal transmittance.

#### 3.2.1. Projected Solid Angle

When the half field angle θ = 0°, the chief ray angle, Θ, in the image space was zero, and the direction cosines of the reference rays R1 and R2 were (0, 0, 1), (0, −0.18469, 0.98280), respectively; then, θ_1_ = cos^−1^(L_1_L_2_ + M_1_M_2_ + N_3_N_2_) = 10.6421°, the numerical aperture NA_0_ = *n*sinθ_1_ = 0.18467, and the numerical aperture was equal in the X- or Y-directions, that is, NA_1_ = NA_X_ = NA_Y_. Therefore, the solid angle $\Omega_{0}=\pi NA_{0}^{2}$ = 0.10714 sr. When the half field angle θ = 32°, the incident angle of the chief ray, R1, in the image space on the curved image plane Θ = 8.54314°, and the direction cosines of reference rays R2 and R3 were (0, 0.31643, 0.94862), (0, 0.56835, 0.82279), respectively. θ_2_ = cos^−1^(L_2_L_3_ + M_2_M_3_ + N_2_N_3_) = 16.18679°; the numerical aperture was NA_Y_ = *n*sin(θ_2_/2) = 0.14079. The direction cosines of reference rays R4 and R5 were (−0.17266, 0.42993, 0.88620) and (0.17266, 0.42993, 0.88620), respectively; θ_3_ = cos^−1^(L_4_L_5_ + M_4_M_5_ + N_4_N_5_) = 19.88447°. Then, the numerical aperture was NA_X_ = *n*sin(θ_3_/2) = 0.17266 and the ordinary solid angle was calculated as follows: Ω_32_ = πNA_X_NA_Y_ = 0.07637 sr; so, the projected solid angle Ω′_32_ = Ω_32_cosΘ′ = 0.07552 sr. Table 4 shows the projected solid angle results for different viewing angles, θ = 0, 5°, 10°, 15°, 20°, 25°, 32°, when the reference wavelength is 587.56 nm.

For reference wavelengths of 656.27, 587.56, and 486.13 nm, the projected solid angles at different half field angles of 0°, 5°, 10°,15°, 20°, 25°, and 32° were calculated; the average values are shown in Table 5.

#### 3.2.2. Surface Transmittance

The curved image plane lens design included three lenses, so there were seven surfaces, including the aperture stop. The third surface was the aperture stop position, located between the first and second lenses, and its s-polarized and p-polarized reflectances were both zero, so they can be ignored, as shown in Table 6. Table 6 also shows the s-polarized reflectance, p-polarized reflectance, un-polarized reflectance, surface transmittances, and total surface transmittances when the reference wavelength is 587.56 nm at a field angle of 0 degrees on each surface of the lens. Table 7 shows the surface transmittance and the average values at different half field angles of 0°, 5°, 10°, 15°, 20°, 25°, and 32° when the wavelengths are 656.27, 587.56, and 486.13 nm.

#### 3.2.3. Internal Transmittance

The lens data used in this study can be obtained from SCHOTT Glass Lens Co., Ltd.(Mainz, Germany). They provided the internal transmittance data for 10 mm thickness lenses at different wavelengths. The internal transmittances obtained at 656.27, 587.56, and 486.13 nm are shown in Table 8.

The three-lens group design consisted of three glass materials. The thickness of the N-FK51A lens was 0.32193 mm, the thickness of the N-SF4 lens was 0.15 mm, and the thickness of the P-SF8 lens was 0.26284 mm. Using the data in Table 9 and Equation (25), the internal transmittance of different lenses can be deduced for a field angle of 0° and at the wavelength of 587.56 nm. Table 10 shows the internal transmittance and average values at different half field angles of 0°, 5°, 10°, 15°, 20°, 25°, and 32° when the wavelengths are 656.27, 587.56, and 486.13 nm.

#### 3.2.4. Calculations and Comparisons of the Relative Illuminance

The relative illuminance can be calculated from the defined projected solid angles, surface transmittances, and internal transmittance. The results are shown in Table 11.

**Figure 9:**
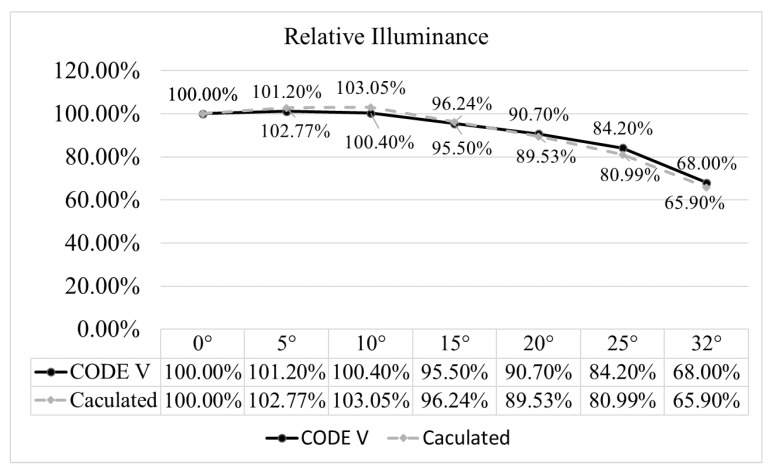
Comparison between the relative illuminance calculated by the formula and the relative illuminance simulated by CODE V software.

To demonstrate the correctness of the results of this study, the relative illuminance was compared with the relative illuminance obtained by the optical software CODE V, as shown in Figure 9. As the results show, the values for the relative illuminance presented in this paper are similar to the simulated values obtained by using the CODE V software.

### 3.3. Image-Quality Analysis of the Three-Lens Design with a Curved Image Plane

**Figure 10:**
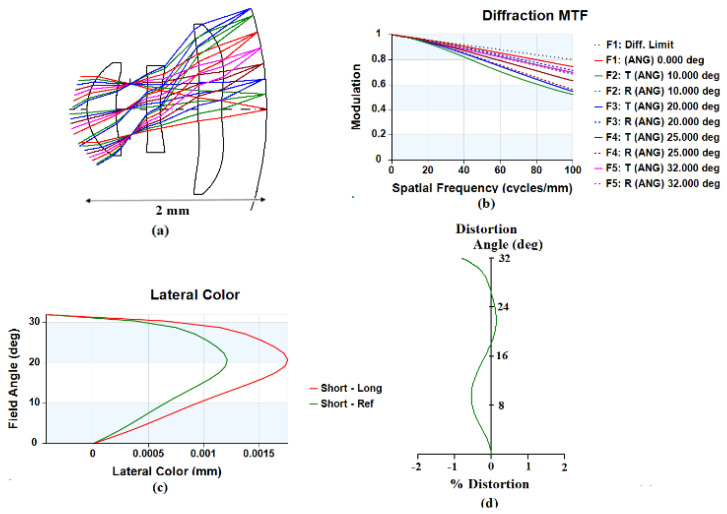
Curved image plane lens design and image-quality analysis: (**a**) three-lens design diagram; (**b**) MTF; (**c**) lateral color; (**d**) optical distortion.

Figure 10a shows the three-lens design with a curved image plane, where the radius of curvature of the curved image plane is *−*3.725 mm. Figure 10b shows the MTF curve diagram, where the minimum value of MTF is 0.52 in the tangential direction with a semi-field angle of 32°. Figure 10c shows the lateral-color curve diagram. The red curve represents the lateral color from the short wavelength (486.1 nm) to the long wavelength (656.3 nm); the maximum lateral color is 2.12 μm. The green curve represents the lateral color from the short wavelength (486.1 nm) to the reference wavelength (587.6 nm); the maximum lateral color is 1.60 μm. Figure 10d shows the optical distortion curve diagram, where the maximum optical distortion is *−*1.01%.

## 4. Conclusions

The relationship between the parameters of lens length and the three-lens design was derived to obtain the short lens length. A shorter back focal length and smaller lens group length were required. The shorter the lens length, the greater the astigmatism and field curvature. However, optimizing the curvature radius of the image plane can reduce the astigmatism and field curvature results, as well as improve the lens quality.

The relative illuminance was related to the projected solid angle of the image plane, the surface transmittance, and the internal transmittance of the three-lens design. We proposed these design methods to achieve the objectives. Using a curved image plane can increase the projected solid angle of the image surface; using an AR coating on the surface of the lens can improve the surface transmittance; and reducing the thickness of the lens can improve the internal transmittance of the lens. The abovementioned three methods can jointly improve the relative illumination of three-lens designs.

## Figures and Tables

**Table 1:** Lens data for the curved image plane design.

Surface No.	Surface Type	Radius (mm)	Thickness (mm)	Glass	Full Aperture
Object		Infinity	Infinity		
1	Asphere	0.60809	0.32193	48,656.84468 (N-FK51A)	1.03479
2	Asphere	1.71796	0.15826		0.86572
Stop		Infinity	0.18291		0.56611
4	Asphere	2.05032	0.15000	75,620.27580 (SF4)	0.78493
5	Asphere	1.48313	0.42407		0.96893
6	Asphere	2.22951	0.26284	68,893.31250 (P-SF8)	1.78978
7	Asphere	1.50771	0.50000		1.89707
Image		−3.72562	0.00000		2.25493

**Table 2:** The aspheric coefficients of the three-lens design.

Surface No.	K	A	B	C	D
1	−5.0530 × 10^−1^	1.5254 × 10^−1^	1.44297	−4.21545	1.3683 × 10^1^
2	3.18955	1.7895× 10^−3^	1.2202 × 10^−1^	−9.9545 × 10^−1^	−5.2982 × 10^−1^
4	2.70553	−6.4165 × 10^−1^	−5.88438	3.1152 × 10^1^	−1.8758 × 10^2^
5	5.65199	−4.7730 × 10^−1^	−1.51130	−2.27032	2.8802 × 10^−1^
6	−1.000 × 10^2^	−5.7731 × 10^−1^	7.0377 × 10^−1^	−2.1970 × 10^−1^	−4.5454 × 10^−2^
7	−8.5819 × 10^1^	−3.9086 × 10^−1^	−5.8701 × 10^−2^	1.4844 × 10^−1^	−7.1665 × 10^−2^

**Table 3:** Relevant parameter data for controlling δ′.

	δ′ = −1.48405 mm		
k	0.50403 (1/mm)	k_12_	0.51154 (1/mm)
d_1_	0.99592 mm	n_3_	1.68893
d_2_	0.73646 mm	t_3_	0.26284 mm
k_1_	0.56600 (1/mm)	k_31_	0.30901 (1/mm)
k_2_	−0.12483 (1/mm)	k_3_	−0.12596 (1/mm)

**Table 4:** Projected solid angles at different field angles with a reference wavelength of 587.56 nm.

θ	Θ	NA_X_	NA_Y_	Ω	Ω′
0	0	0.18469	0.18467	0.10714 sr	0.10714 sr
5°	5.24354°	0.18630	0.18894	0.11058 sr	0.11012 sr
10°	9.79328°	0.18702	0.19080	0.11210 sr	0.11047 sr
15°	13.71356°	0.18461	0.18330	0.10630 sr	0.10327 sr
20°	16.20176°	0.18099	0.17599	0.10007 sr	0.09609 sr
25°	15.66270°	0.17740	0.16322	0.09097 sr	0.08759 sr
32°	8.54314°	0.17266	0.14080	0.07637 sr	0.07552 sr

**Table 5:** Projected solid angles at different reference wavelengths and field angles.

Half Field Angle θ	Projected Solid Angle			
	656.27 nm	587.56 nm	486.13 nm	Average
0°	0.10638	0.10717	0.10862	0.10739
5°	0.10931	0.11012	0.11163	0.11035
10°	0.10973	0.11047	0.11184	0.11068
15°	0.10268	0.10327	0.10435	0.10344
20°	0.09563	0.09609	0.09695	0.09622
25°	0.08730	0.08759	0.08815	0.08768
32°	0.07534	0.07552	0.07596	0.07561

**Table 6:** Surface transmittances of each surface with a wavelength of 587.56 nm and field angle of 0 degrees.

Surface No.	Glass Material	s-Polarized Reflectance	p-Polarized Reflectance	un-Polarized Reflectance	Surface Transmittance
1	N-FK51A	0.01518	0.01518	0.01518	0.98482
2	air	0.01518	0.01518	0.01518	0.98482
4	SF4	0.00166	0.00166	0.00166	0.99834
5	air	0.00166	0.00166	0.00166	0.99834
6	P-SF8	0.00360	0.00360	0.00360	0.99640
7	air	0.00360	0.00360	0.00360	0.99640
Total					0.95970

**Table 7:** Surface transmittances and average values at different wavelengths and field angles.

Half Field Angle	Surface Transmittance			
	656.27 nm	587.56 nm	486.13nm	Average
0°	0.95010	0.95970	0.92751	0.94569
5°	0.94998	0.95969	0.92775	0.94573
10°	0.94964	0.95950	0.92789	0.94560
15°	0.94882	0.95882	0.92731	0.94491
20°	0.94667	0.95710	0.93105	0.94490
25°	0.93938	0.95161	0.92347	0.93810
32°	0.88188	0.89806	0.87549	0.88514

**Table 8:** Internal transmittances measured for a 10 mm sample thickness.

Glass Materials	Internal Transmittance		
Wavelength	656.27 nm	587.56 nm	486.13 nm
N-FK51A	0.9980	0.9980	0.9980
N-SF4	0.9980	0.9980	0.9965
P-SF8	0.9940	0.9940	0.9892

**Table 9:** Internal transmittances for a wavelength of 587.56 nm and a field angle of 0°.

Lens No.	Glass Materials	Thickness (mm)	Internal Transmittance
1	N-FK51A	0.32193	0.99994
2	N-SF4	0.15000	0.99997
3	P-SF8	0.26284	0.99984
Total			0.99975

**Table 10:** Internal transmittances and average at different wavelengths and field angles.

Semi Field Angle	Internal Transmittance			
	656.27 nm	587.56 nm	486.13 nm	Average
0°	0.99975	0.99975	0.99960	0.99970
5°	0.99981	0.99989	0.99940	0.99970
10°	0.99981	0.99989	0.99938	0.99969
15°	0.99981	0.99988	0.99937	0.99969
20°	0.99981	0.99982	0.99936	0.99966
25°	0.99981	0.99988	0.99938	0.99969
32°	0.99981	0.99984	0.99963	0.99976

**Table 11:** Relative illuminances and averages at different wavelengths and field angles.

Semi Field Angle	Relative Illuminance			
	656.27 nm	587.56 nm	486.13 nm	Average
0°	100.00%	100.00%	100.00%	100.00%
5°	102.75%	102.77%	102.78%	102.77%
10°	103.10%	103.07%	102.99%	103.05%
15°	96.40%	96.29%	96.03%	96.24%
20°	89.57%	89.43%	89.57%	89.53%
25°	81.14%	81.05%	80.78%	80.99%
32°	65.74%	65.95%	66.01%	65.90%

## Data Availability

Data are contained within the article.
